# Association between sexually transmitted infections and reproductive lifespan: analysis of the NHANES 1999–2023

**DOI:** 10.1186/s12889-026-27795-2

**Published:** 2026-05-22

**Authors:** Yingjing Wu, Shuping Yang, Qi Ding, Tingting Hao, Yu Zhu, Yue e Huang, Yali Liang

**Affiliations:** School of Public Health, Wannan Medical College, 22 Wenchang West Road, Yijiang District, Wuhu, Anhui Province China

**Keywords:** Sexually transmitted infections, Reproductive lifespan, NHANES, Reproductive health

## Abstract

**Background:**

Reproductive lifespan (RLS), defined as the interval from menarche to natural menopause, serves as a comprehensive indicator of a woman’s reproductive capacity. Sexually transmitted infections (STIs) induce various outcomes, including accelerated ovarian aging, miscarriage, pregnancy loss, preterm birth, sexually tract microenvironmental dysregulation and decreased ovarian reserve markers Anti-Mullerian hormone (AMH). However, there is currently a rareness of population-bases epidemiological studies on the relationship between STIs and RLS. This study aimed to evaluate the association between STIs and RLS among females in the United States.

**Methods:**

Using data from the National Health and Nutrition Examination Survey (NHANES) 1999–2023. Multiple linear regression models were constructed to assess the association between STIs and RLS. Interaction tests and subgroups analyses were conducted to assess heterogeneity across different subgroups.

**Results:**

The analysis included a nationally representative cohort of 1,715 women. After adjusting for potential confounding factors, HPV infection positive was significantly associated with a shorter RLS, corresponding to a reduction of 0.65 years (95% CI: -1.22, -0.07; *P* = 0.027). High-risk HPV was negatively associated with RLS (β= -1.26,95%CI: -2.04, -0.49, *p* = 0.001), indicating that higher HPV infection was associated with a shorter RLS, while non-high-risk HPV infection positive was associated with a reduction of 1.03 years (95% CI: -1.75, -0.32; *P* = 0.005). Subgroup analysis reveals high-risk HPV consistently showed stronger associations with shorter RLS, with interaction effects observed mainly for educational level (P for interaction = 0.018) and marital status (P for interaction < 0.001). Unfortunately, no significant associations were observed between RLS and other infections, including HSV-2, BV, TV, GC, and CT.

**Conclusions:**

Our findings reveal that STIs impair female reproductive potential, with HPV infection in particular significantly shortening RLS. Prevent and control STIs will bring benefits to human reproductive health.

**Supplementary Information:**

The online version contains supplementary material available at 10.1186/s12889-026-27795-2.

## Background

 Reproductive health is a fundamental component of overall human health, shaping fertility, pregnancy outcomes, and lifelong physical and psychological well-being [1]. A key indicator of female reproductive health is the reproductive lifespan (RLS), defined as the interval between menarche and menopause, which serves as an integrated indicator of ovarian function, endocrine status and the aging of reproductive organs in females [2]. It reveals intricate health risk interactions across life stages [3]. Shorter RLS has been associated with increased risks of infertility, cardiovascular disease, osteoporosis, and all-cause mortality [4–7]. Generally speaking, the female RLS falls within a certain natural range, and deviations below this range can lead to varying degrees of adverse effects on women’s health as well as the well-being of future generations. Under typical demographic conditions, childbearing during a woman’s prime reproductive years serves as the primary driver of population trends and expansion [8]. However, the biological and environmental determinants of RLS remain incompletely understood.

Sexually transmitted infections(STIs) are increasingly recognized as a serious global health burden with impact on individual women and a key threat to female reproductive well-being [9]. The World Health Organization (WHO) reported that in 2020 the global annual infection people of common STIs among individuals aged 15–49 years were approximately 3.4% for Chlamydia trachomatis (CT), 2.2% for gonorrhoeae (GC), 4.1% for Trichomonas vaginalis (TV) [10, 11]. These three infections are curable, unlike viral infections such as Human Immunodeficiency Virus(HIV), herpes simplex virus type 2 (HSV-2) and human papillomavirus (HPV), requiring long term management and treatment [12].Among chronic infections, the prevalence rate of HSV-2 reached 13%, while bacterial vaginosis (BV) affected 23–29%. Furthermore, HPV remains the most widespread STI worldwide, infecting roughly 23% women and is often studied alongside STIs due to its overlapping risk factors and reproductive health implications.

Prior evidence has indicated that multiple sexually tract pathogens may induce chronic inflammatory responses, immune dysregulation, and structural damage to reproductive tissues, ultimately leading to adverse reproductive outcomes [13, 14]. HPV, the most prevalent genital tract pathogen, is well established as the primary etiologic agent of cervical cancer [15, 16]. Furthermore, emerging evidence has suggested that HPV infection may influence reproductive function in addition to oncogenesis [17]. Biologically, HPV may impair reproductive capacity through involving chronic cervical inflammation, dysregulating local immunity, altering follicular signaling, and disruption of the reproductive tract microenvironment [18]. A case-control study has reported that HPV infection is associated with altered cervical cytokine profiles, decreased ovarian reserve markers such as AMH, and higher risks of subfertility [19]. HSV-2 infection has been associated with elevated risks of miscarriage and preterm birth [20].

For instance, CT and GC are primary causes of pelvic inflammatory disease and are closely associated with fallopian tube obstruction, ectopic pregnancy, and secondary infertility [21]. In addition, common lower genital tract disorders such as TV infection and BV can disrupt the vaginal microbiota, promote mucosal inflammation, and increase the probability of adverse pregnancy outcomes [11, 13]. Collectively, these infections possess the potential to induce persistent inflammation, chronic immune activation, and local hormonal signaling disruption, thereby impairing ovarian and uterine function. This suggests they may play a significant role in regulating RLS [22].

Most existing studies have focused on single pathogens and short-term reproductive outcomes, such as infertility or pregnancy loss. The cumulative impact of multiple STIs on the RLS has seldom been investigated. Our study aims to evaluate the associations between six common STIs—HPV, HSV-2, BV, TV, CT, GC and RLS. These STIs underscore the importance of comprehensive prevention, screening, and control strategies. By integrating pathogen-specific exposures with reproductive aging outcomes, our study aims to explore relationship between STIs and RLS, then provide evidence that may help extend women’s reproductive years and support future strategies for reproductive health prevention.

## Materials and methods

### Study population

**Fig. 1:**
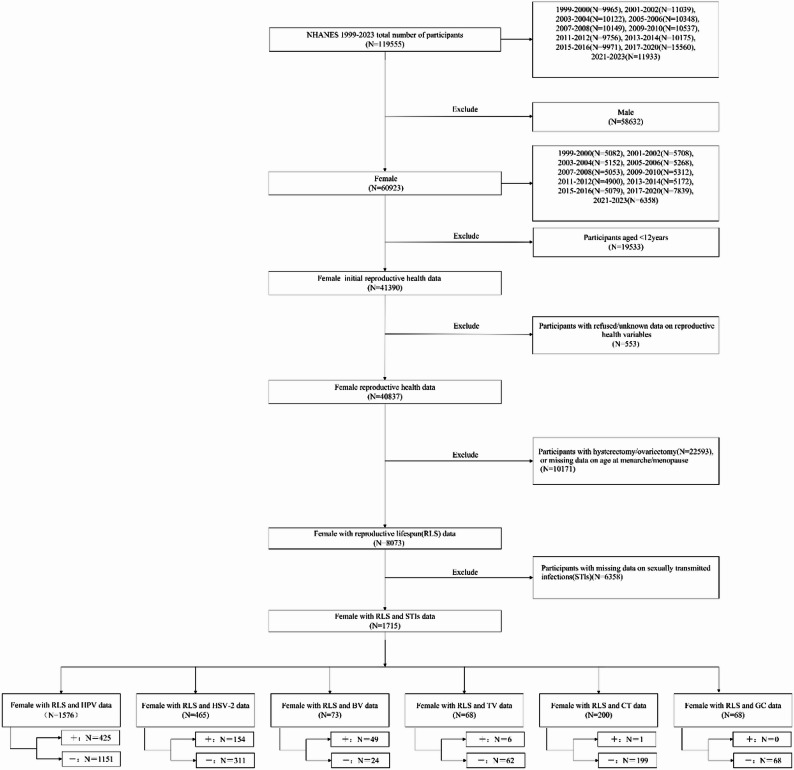
Flowchart of the participant selection process between HPV and female reproductive lifespan of the NHANES Survey (1999–2023)

This cross-sectional study utilized publicly available data from the National Health and Nutrition Examination Survey (NHANES). All procedures followed in the NHANES study were reviewed and approved by an institutional ethics board, and participation was voluntary, with written informed consent obtained from all individuals prior to data collection. From 1999 to 2023, a total of 119,555 samples participated in the NHANES study. Participants were excluded according to the following criteria: (1) males (*N* = 58,632); (2) age under 12 years (*N* = 19,533); (3) Participants with refused or unknown data on reproductive health variables (*N* = 553); (4) Participants who had undergone hysterectomy or ovariectomy (*N* = 22,593); missing age at menarche or menopause data (*N* = 10,171). A total of 8,073 participants had reproductive health data that met the criteria. The study ultimately 1,715 participants have STIs and RLS valid data were included in analysis. The flow chart of the screening process is shown in Fig.1.

Laboratory measurement: serological testing for HPV, HSV-2, BV, TV, CT and GC 

HPV infection was detected using the Roche Linear Array HPV Genotyping Test, which identifies 37 (including types 6, 11, 16, 18, 26, 31, 33, 35, 39, 40, 42, 45, 51, 52, 53, 54, 55, 56, 58, 59, 61, 62, 64, 66, 67, 68, 69, 70, 71, 72, 73, 81, 82, 83, 84, 89, and IS39) HPV genotypes [23]. High-risk HPV types include 16, 18, 31, 33, 35, 39, 45, 51, 52, 56, 58, 59, 68, 73, 82, non-high risk HPV types include 6, 11, 26, 40, 42, 53, 54, 55, 61, 62, 64, 66, 67, 69, 70, 71, 72, 81, 83, 84, 89, and IS39. A positive result for any type was classified as an HPV infection. Seropositivity for HSV-2 was determined using a solid-phase enzyme immunoassay targeting type-specific glycoprotein G-2 (gG-2) antibodies [24]. BV was diagnosed via Gram-stained vaginal smears evaluated by the Nugent scoring system (0–10). A score of 7–10 was defined as BV [25]. TV was detected by PCR amplification of a 312-bp fragment of the 18 S rRNA gene, with product confirmation by Southern blot hybridization using a digoxigenin-labeled probe. Sample adequacy was verified by concurrent β-globin PCR [26]. CT and GC were detected using the BD Probe Tec ET system, which employs Strand Displacement Amplification (SDA) to target pathogen-specific DNA. Results were determined by comparing the fluorescent MOTA score to a pre-defined cutoff [27].

### RLS

The RLS was defined as the interval between the age at menarche and the age at natural menopause [28]. Natural menopause refers to menopause that occurs for natural reasons within the past 12 months, and who had not undergone any ovary removed or hysterectomy before their last menstrual period [29].

Covariates:

Covariates included age, race, education level, poverty/income ratio (PIR), body mass index (BMI), physical activity level (PA), number of pregnancies, first sexual age, number of sexual partners, marital status, Smoking status, Hypertension, Diabetes and Female hormone use. Age was dichotomized by < 40 years and ≥ 40 years [30]; Educational level was grouped into two categories: ≤high school and > high school [30]. PIR was split into two groups of under 1.3 and 1.3 or higher [31]. BMI was divided into three categories: Normal weight (18.5–24.9), Underweight (< 18.5) and Overweight (> 25) [32]. Physical activity levels (PA) were estimated using metabolic equivalent (MET) scores assigned to each activity based on its type and intensity—8.0 for vigorous, and 4.0 for both medium and light activities. For each reported activity, we multiplied its MET value by how often and how long it was done over the past 30 days or week, giving MET-minutes per 30 days or per week. We then added up these values across all activities. For those who provided monthly data, we converted their total MET-minutes per 30 days into a weekly estimate by dividing by 30 and multiplying by 7. Finally, participants were grouped into three activity levels following the WHO 2020 guidelines [33]: light (< 600 MET-min/week), medium (600–1,200 MET-min/week), and vigorous (≥ 1,200 MET-min/week) [34]. Number of pregnancies was categorized into two groups: <3 times and ≥ 3 times [35]; First sexual age was categorized into two groups: <18 years, ≥ 18 years [36]; Number of sexual partners was categorized into two groups: ≤1 and > 1 [37]; Smoking status was classified into three groups based on lifetime use and current behavior: never smoked, reported smoking fewer than 100 cigarettes in their lifetime, former smoker, had smoked at least 100 cigarettes in life but did not smoke currently and current smoker, currently smokes, whether daily or occasionally, and has smoked at least 100 cigarettes total [38].

### Statistical analysis

Missing covariate included the PIR (7% missing), first sexual age (6% missing), and number of sexual partners lifetime (8% missing). Missing covariate data were handled using multiple imputation via chained equations (MICE). 20 imputed datasets were generated using the MICE package in R. Continuous variables were imputed using predictive mean matching, and categorical variables were imputed using logistic or polytomous regression models. The pooled estimates were calculated using Rubin’s rules. Continuous variables (RLS, age, PIR, BMI, Mets, number of pregnancies, first sexual age, number of sexual partners lifetime) were presented as Medians (interquartile ranges) and compared using Mann-Whitney U test. The categorical variables (race, educational level, marital status, smoking status, Hypertension, Diabetes, Female hormone use) were expressed as numbers (percentages), compared with the chi-square test or fisher exact. HPV, HSV-2, BV, TV, GC, CT was treated as a categorical variable. Multiple linear regression models were constructed to assess the association between HPV, HSV-2, BV, TV, GC, CT and RLS. In addition, a linear regression was performed using high-risk HPV and non-high-risk HPV as categorical independent variables to further assess the relationship with RLS. Besides, we performed stratified and interaction analyses for all covariates.

All analyses were performed with R (version 4.4.2) and SPSS (version 27.0). A result was considered statistically significant if the p-value was less than 0.05.

## Result

### Baseline characteristics of the participants

Table S1 summarizes the baseline characteristics of the study population stratified by HPV and HSV-2 infection status. The overall HPV infection positive rate was 26.95% (424/1573). Of these, high-risk HPV genotypes were identified in 190 cases, accounting for 44.81% of the HPV-positive individuals. The median RLS was 33 years, with an interquartile range of 26 to 37 years in the HPV-positive group. HPV infection positive was significantly younger than those without (53 vs. 54, *P* < 0.001), had a lower PIR, and had an earlier first sexual age. They also reported a higher number of lifetime men sexual partners. 33.12% of whom 154/465 were HSV-2 infection positive. HSV-2 infection positive group was older (45.5 vs. 41, *P* < 0.001), had a lower PIR, an earlier sexual age, and more sexual partners. Significant differences in race distribution were observed for both HPV (*P* < 0.001) and HSV-2 (*P* < 0.001), with a notably higher proportion of Non-Hispanic Black individuals in both infection positive groups. Marital status also differed significantly between groups for both infections (*P* < 0.001), with lower proportions of married individuals among both infection positive participants.

The HPV- and HSV-2-infected groups had a higher proportion of current smokers than the non-infected group (HPV: 32.47% vs. 19.46%; HSV-2: 35.06% vs. 22.19%; both *P* < 0.01). However, HSV-2-positive participants had significantly lower educational level (51.95% vs. 41.16% with ≤high school education, *P* = 0.028) and higher number of pregnancies (3 vs. 2.21, *P* = 0.001). No significant differences were observed in educational level, hypertension, diabetes or female hormone use in either infection group. BMI was lower in the HPV-infection positive group (28.70 vs. 29.82, *P* = 0.004) but did not differ by HSV-2 group. For other STIs, 67.12% of BV-positive, with a statistically significant difference in METs between the two groups (see Table S2). 6 out of 68 participants (8.82%) were TV-positive, with a significant difference in educational level between groups (see Table S3). Out of 200 participants, only 1 tested positive for CT (see Table S4). No GC-positive was detected among the 68 participants.

### Linear regression analysis of the association between HPV infection status and RLS (years)

A significant association between HPV infection status and RLS was observed in the linear regression analyses presented in Table 1. HPV was analyzed both as a binary variable, showing a consistent negative significantly association with RLS across all models. In the fully adjusted Model 3, HPV infection positive was significantly associated with a shorter RLS, corresponding to a reduction of 0.65 years (95% CI: -1.22, -0.07; *P* = 0.027). When HPV was subsequently categorized into three classifications (Negative as the reference), with full covariate adjustment in Model 3, high-risk HPV infection positive was associated with a significant reduction in RLS of -1.26 years (95% CI: -2.04, -0.49; *P* = 0.001), while non-high-risk HPV infection positive was associated with a reduction of -1.03 years (95% CI: -1.75, -0.32; *P* = 0.005).

**Table 1:** Linear regression analysis of the association between HPV infection status and RLS (years) across different models

Exposure	Model1		Model2		Model3	
	β (95%CI)	*P*	β (95%CI)	*P*	β (95%CI)	*P*
HPV						
Negative	Reference		Reference		Reference	
Positive	-3.06 (-4.04 ~ -2.08)	**< 0.001**	-0.96 (-1.53 ~ -0.39)	**< 0.001**	-0.65 (-1.22 ~ -0.07)	**0.027**
High risk	-3.82 (-4.95 ~ -2.69)	**< 0.001**	-1.58 (-2.34 ~ -0.82)	**< 0.001**	-1.26 (-2.04 ~ -0.49)	**0.001**
Non high risk	-1.61 (-2.66 ~ -0.56)	**0.003**	-1.27 (-1.98 ~ -0.56)	**< 0.001**	-1.03 (-1.75 ~ -0.32)	**0.005**

*CI* Confidence Interval

Model1: Crude

Model2: Adjust: race, age

Model3: Adjust: race, educational level, marital status, smoking status, hypertension, diabetes, female hormone use, age, PIR, BMI, Mets, number of pregnancies, first sexual age, number of sexual partners

*Values in bold indicate statistical significance (*p* < 0.05)

### Linear regression analysis of the association between HSV-2, BV, TV and CT infection status and RLS (years)

The results of linear regression analyses examining the association between HSV-2, BV, TV and CT infection status and RLS was presented in Table 2. In the crude Model 1, only HSV-2 infection positive was associated with a significant increase in RLS of 3.19 years (95% CI: 1.28, 5.10; *P* = 0.001). Specifically in the fully adjusted Model 3, there was no significant association in HSV-2, BV, TV and CT infection and RLS.

**Table 2:** Linear regression analysis of the association between HSV-2, BV, TV and CT infection status and RLS (years)

Exposure	Model1		Model2		Model3	
	β (95%CI)	*P*	β (95%CI)	*P*	β (95%CI)	*P*
HSV-2						
Negative	Reference		Reference		Reference	
Positive	3.19 (1.28 ~ 5.10)	**0.001**	-0.39(-1.25 ~ 0.48)	0.382	-0.48(-1.43 ~ 0.46)	0.318
BV						
Negative	Reference		Reference		Reference	
Positive	-0.33 (-5.27 ~ 4.61)	0.896	-0.90(-2.40 ~ 0.61)	0.246	-0.73(-2.35 ~ 0.88)	0.379
TV						
Negative	Reference		Reference		Reference	
Positive	5.44(-3.00 ~ 13.88)	0.211	2.12 (-0.32 ~ 4.55)	0.094	0.22(-2.98 ~ 2.53)	0.876
CT						
Negative	Reference		Reference		Reference	
Positive	-8.01(-19.43 ~ 3.41)	0.171	-3.71(-9.65 ~ 2.24)	0.223	-2.28(-8.39 ~ 3.89)	0.474

*CI* Confidence Interval

Model1: Crude

Model2: Adjust: race, age

Model3: Adjust: race, educational level, marital status, smoking status, hypertension, diabetes, female hormone use, age, PIR, BMI, Mets, number of pregnancies, first sexual age, number of sexual partners

*Values in bold indicate statistical significance (*p* < 0.05)

## Subgroup analysis

### Subgroup analysis of the association between HPV infection status and RLS (years)

**Fig. 2:**
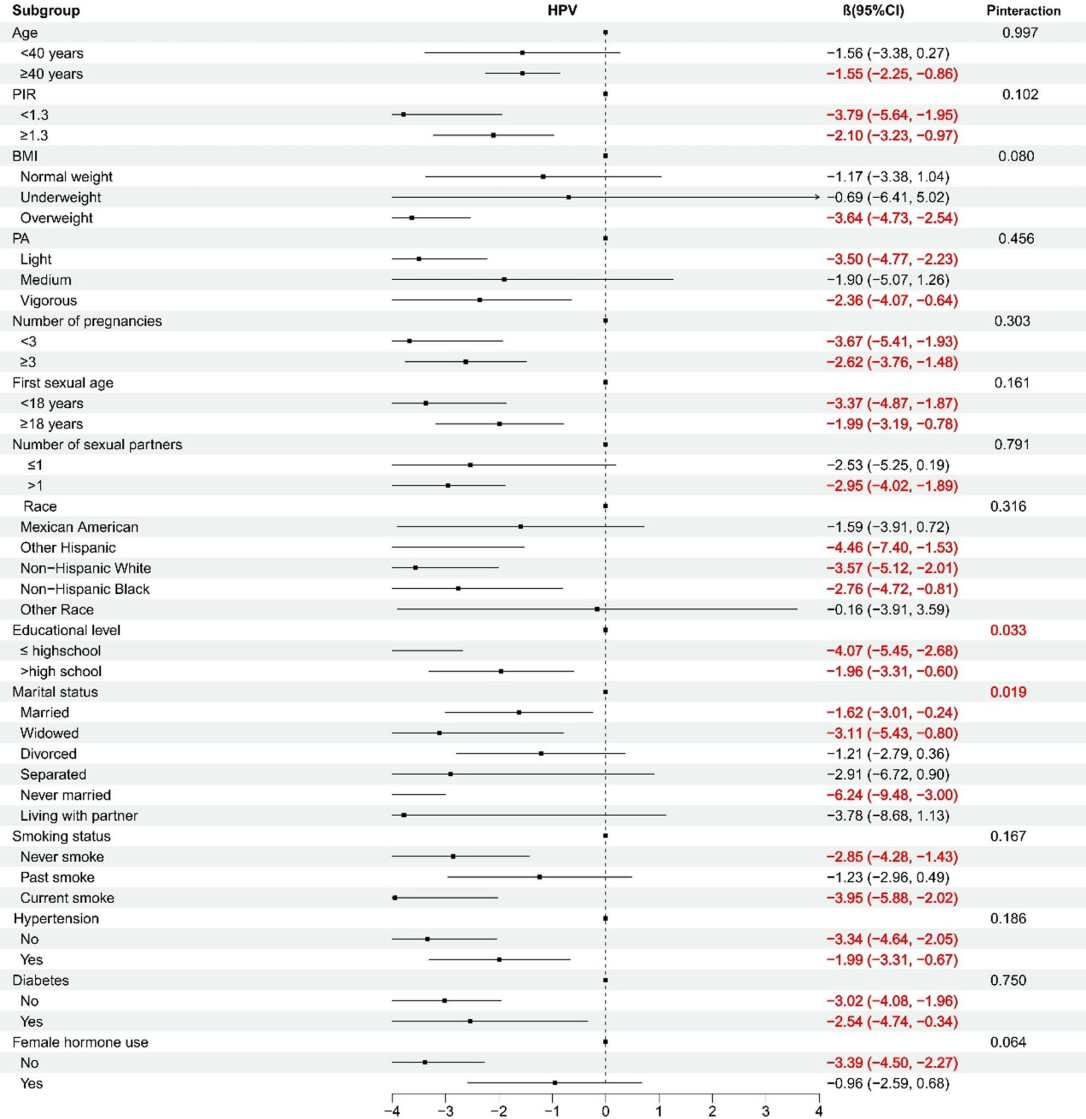
Stratified Analyses by Potential Modifiers of the Association between HPV and RLS. *Each subgroup analysis adjusted for age, race, PIR, BMI, PA, number of pregnancies, first sexual age, number of sexual partners, educational level, marital status, smoking status, hypertension, diabetes, female hormone use, except for the stratifying variable

Stratified analyses were performed to examine the association between HPV infection status and RLS across subgroups of age, PIR, BMI, PA, number of pregnancies, first sexual age, number of sexual partners, race, educational level and marital status, smoking status, Hypertension, Diabetes and female hormone use (Fig. 2).

Specifically, the inverse association between HPV and RLS was more pronounced in individuals with lower educational level (≤ High school: β = -4.07, 95% CI: -5.45, -2.68) than in those with higher education (> High school: β = -1.96, 95% CI: -3.31, -0.60). Regarding marital status, the strongest negative association was observed in never-married participants (β = -6.24, 95% CI: -9.48, -3.00), while married individuals showed a more modest association (β = -1.62, 95% CI: -3.01, -0.24).

Significant effect modifications were observed for educational level (P for interaction = 0.033) and marital status (P for interaction = 0.019). Only in marital status and educational level with stronger effects observed in socially disadvantaged groups, indicating potential health disparities in HPV-related reproductive health outcomes. Notably, the negative association remained consistent across most age groups, though it was statistically significant only in participants aged ≥ 40 years, overweight individuals and those with lower PIR.

### Subgroup analysis of the association between risk stratification of HPV infection status and RLS (years)

**Fig. 3:**
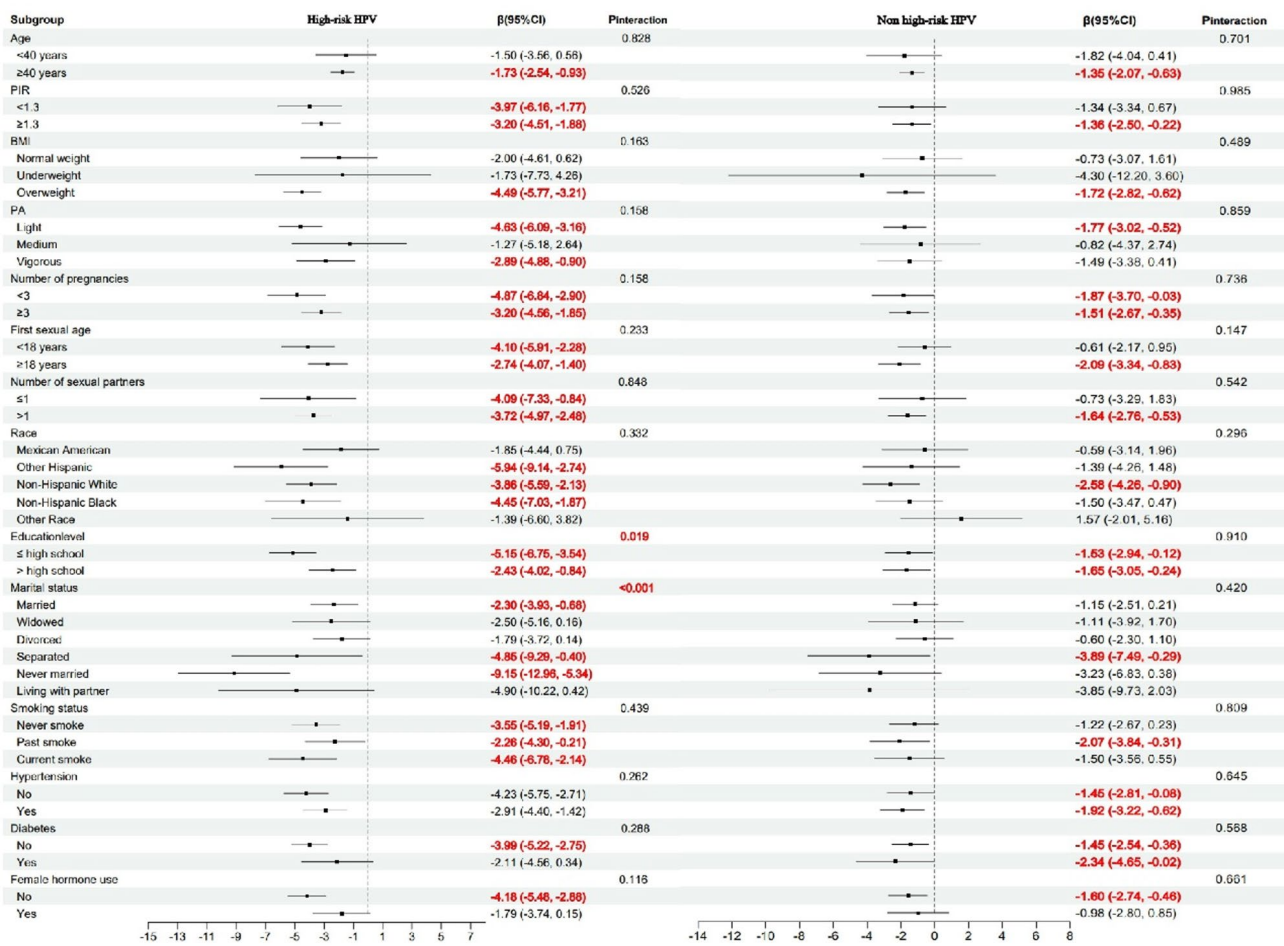
Stratified Analyses by Potential Modifiers of the Association between HPV and RLS by high-risk HPV and non-high-risk HPV. *Each subgroup analysis adjusted for age, race, PIR, BMI, PA, number of pregnancies, first sexual age, number of sexual partners, educational level, marital status, smoking status, hypertension, diabetes, female hormone use, except for the stratifying variable

Figure 3 presents the results of stratified analyses evaluating the association between risk stratification of HPV infection status and RLS. A consistent inverse association was observed between both high-risk and non-high-risk HPV infection and RLS in several key subgroups, including participants aged ≥ 40 years and those who were overweight. However, the pattern of this association and the presence of effect modification differed markedly between the two HPV risk categories. For high-risk HPV infection, the strength of the inverse association was significantly modified by specific socioeconomic factors. A notably stronger association was observed in individuals with ≤ high school (β = -5.15, 95% CI: -6.75, -3.54) compared to those with > high school (β = -2.43, 95% CI: -4.02, -0.84; P for interaction = 0.019). Similarly, marital status was a significant effect modifier (P for interaction < 0.001), with the most substantial association found in never-married participants (β = -9.15, 95% CI: -12.96, -5.34) compared to their married counterparts (β = -2.30, 95% CI: -3.93, -0.68).

Crucially, and in direct opposition to the findings for high-risk HPV, none of the examined variables demonstrated a significant effect modification (all P for interaction > 0.05).

In summary, while both HPV risk types are inversely associated with RLS, high-risk HPV infection exhibits a stronger, more concentrated effect that is modified by socioeconomic vulnerability (e.g., lower education and being never-married). Non-high-risk HPV infection, however, appears to have a broader, more generalized association with RLS that is not preferentially modified by any of the demographic or clinical factors assessed in this study.

## Discussion

STIs were negatively associated with RLS. Specifically, HPV infection was associated with a 0.65-year reduction in RLS, while high-risk HPV was linked to a 1.26-year decrease in reproductive lifespan. Subgroup analyses further revealed that the negative impact of HPV infection on RLS was more pronounced among women with lower educational level and never married status.

It is widely recognized that HPV is the primary risk factor for cervical cancer and also causing vulvar and vaginal cancers, significantly impacting women’s fertility and reproductive health. These HPV-associated tumors pose substantial threats to women’s fertility and overall reproductive health. In contrast, no significant associations were observed between RLS and other infections, including HSV-2, BV, TV, GC, and CT. These findings highlight HPV as a potential determinant of reproductive aging, suggesting that viral persistence and its immunological consequences may contribute to earlier reproductive senescence [17, 39]. The observed negative association between HPV and RLS aligns with accumulating evidence that chronic viral infection can alter ovarian endocrine function and accelerate reproductive aging [40, 41]. A case-control study have reported that HPV infection is associated with altered cervical cytokine profiles, decreased ovarian reserve markers such as AMH, and higher risks of subfertility [19]. A review suggests primordial follicles are the starting point of follicular development and the basic functional unit of female reproduction [42]. HPV may also cause systemic inflammatory mediators may traverse the blood-ovarian barrier, disrupting signalling pathways that maintain primordial follicles in a quiescent state (such as the PTEN/PI3K/Akt pathway). This directly leads to their premature and excessive activation and impair granulosa cell function and lower serum AMH levels [17]. Thereby accelerating reserve depletion and thus determines the length of female RLS. Supported by other studies, HPV infection may reduce reproductive potential, thereby shortening RLS. High-risk HPV types can induce recurrent infections that perturb the cervical-vaginal microenvironment and potentially affect the hypothalamic–pituitary–ovarian axis through immune-endocrine crosstalk. Subgroup analyses further revealed that the negative impact of HPV infection on RLS was more pronounced among women with lower educational attainment and never married status. Women may act more liberally sexually before settling down in a stable relationship. Moreover, HPV infection rates are higher in married, separated, and never married women, never married women may have a higher chance to have a risky sexual partner or multiple sexual partners in these groups [43]. Women with higher education levels had lower HPV infection rates, likely because women with higher educational levels pay more attention to their health and maintain good personal life and hygiene habits [44]. These findings underscore the intersection between biological and social determinants of reproductive aging. High-risk HPV infections, in particular, exhibited the strongest effects in socioeconomically disadvantaged groups, suggesting potential cumulative vulnerability arising from delayed diagnosis and persistent infection. Chronic local inflammation induced by persistent HPV infection may extend beyond the cervix and uterus to trigger low-grade systemic immune activation, thereby accelerating ovarian follicle depletion through inflammatory and oxidative damage to granulosa cells and follicular microenvironment. This process may hasten the decline of ovarian reserve, resulting in earlier reproductive senescence [45, 46]. Concurrent viral infection may interfere with steroid hormone metabolism or disrupt feedback mechanisms along the hypothalamic–pituitary–gonadal axis (HPG) [47]. Perturbations in gonadotropin secretion or sex steroid homeostasis can impair follicular maturation [48], shorten the duration of ovulatory cycles across the lifespan, and ultimately reduce RLS [49, 50]. Additionally, HPV-related microbiome alteration could disturb mucosal immune equilibrium, leading to recurrent inflammation and local oxidative stress within the reproductive tract [51]. Increased oxidative burden and inflammatory signaling have been implicated in accelerated follicular atresia and oocyte quality decline, a compromised mucosal barrier and a pro-inflammatory environment may not only facilitate viral persistence but also create a hostile setting for gamete function and implantation, indirectly reducing fecundability and contributing to a cumulative decline in reproductive function [52]. Epigenetic modifications triggered by chronic viral challenges may alter gene expression programs in ovarian or endometrial tissues, including pathways related to folliculogenesis, DNA repair, mitochondrial stability, and steroidogenesis [53, 54]. Such epigenomic reprogramming could produce long-lasting impacts on ovarian aging trajectories, thereby influencing RLS at the tissue level [55]. A review has found the expression of high-risk HPV oncoproteins (E6/E7) may directly induce cellular senescence in ovarian granulosa or theca cells [56]. Senescent cells secrete abundant inflammatory factors, chemokines, and matrix-degrading enzymes, forming a senescence-associated secretory phenotype (SASP). The SASP creates a pro-inflammatory and pro-fibrotic “toxic” microenvironment within the ovary, which accelerates the functional decline and atresia of adjacent healthy follicles [57].

Together, persistent viral exposure—particularly HPV—promotes chronic inflammation, endocrine disturbance, microbial dysbiosis, and epigenetic reprogramming, cumulatively accelerating ovarian aging and leading to a reduced RLS. Therefore, we need to provide more epidemiologic evidence for developing targeted pathogen screening, subtype-specific management, and early intervention strategies to preserve long-term reproductive health.

While leveraging a nationally representative cohort, robust covariate adjustment and comprehensive evaluation of multiple genital tract pathogens. Moreover, by distinguishing between high-risk and non-high-risk HPV genotypes, this study provides novel insight into differential infection effects on reproductive aging. However, several limitations should be acknowledged. First, the cross-sectional design precludes causal inference and reliance on retrospectively reported RLS estimation introduces potential recall bias. Besides, HPV infection status was assessed at a single time point, which cannot fully capture chronic or recurrent exposure. Finally, due to data unavailability, important variables such as a history of pelvic inflammatory disease (PID), HPV vaccination status, and genetic susceptibility could not be adjusted for, which remains a limitation and a potential source of residual confounding. Further longitudinal studies are needed to confirm these observations and explore the mechanisms involved.

## Conclusion

In conclusion, STIs impair female reproductive potential, with HPV infection in particular significantly shortening RLS. Prevent and control STIs brings will benefit to human reproductive health.

## Supplementary Information

Supplementary Material 1.

Supplementary Material 2.

Supplementary Material 3.

Supplementary Material 4.

## Data Availability

Publicly available datasets were analyzed in this study. This data can be found here: [https://wwwn.cdc.gov/nchs/nhanes/](https:/wwwn.cdc.gov/nchs/nhanes) .

## Abbreviations

RLS: Reproductive lifespan
STIs: Sexually transmitted infections
AMH: Anti-Mullerian hormone
WHO: The World Health Organization
CT: Chlamydia trachomatis
GC: Gonorrhoeae
TV: Trichomonas vaginalis
HIV: Human Immunodeficiency Virus
HSV-2: Herpes simplex virus type 2
HPV: Human papillomavirus
BV: Bacterial vaginosis
NHANES: National Health and Nutrition Examination Survey
gG-2: Glycoprotein G-2
SDA: Strand Displacement Amplification
PIR: Poverty/income ratio
BMI: Body mass index
PA: Physical activity
MET: Metabolic equivalent
HPG: Hypothalamic–pituitary–gonadal
SASP: Senescence-associated secretory phenotype
PID: Pelvic inflammatory disease
