# Supplementary material for: Association between sexually transmitted infections and reproductive lifespan: analysis of the NHANES 1999–2023
Source: BMC Public Health. 2026 May 22;26:2161. doi: 10.1186/s12889-026-27795-2 (PMC13374277; doi:10.1186/s12889-026-27795-2)
Supplement: Supplementary file 1 — Supplementary Material 1. [file 12889_2026_27795_MOESM1_ESM.docx]

***Table S1 Baseline characteristics of the HPV and HSV-2 participants***

| Characteristics | Total  (n = 1573) | HPV | | Z/χ² | P | Total  (n = 465) | HSV-2 | | Z/χ² | P |
| --- | --- | --- | --- | --- | --- | --- | --- | --- | --- | --- |
|  |  | Negative  (n = 1149) | Positive  (n = 424) |  |  |  | Negative  (n = 311) | Positive  (n = 154) |  |  |
| RLS | 35 (30, 38) | 35 (31, 38) | 33 (26, 37) | -5.51 | **<.001** | 24 (14, 32) | 23 (12, 32) | 26 (18, 33) | -2.91 | **0.004** |
| Age | 53 (49, 56) | 54 (50, 57) | 52 (47, 56) | -4.25 | **<.001** | 43 (30, 47) | 41 (28, 47) | 45.50  (35.25, 48) | -4.04 | **<.001** |
| PIR | 2.34  (1.15, 4.70) | 2.58  (1.24, 4.96) | 1.75  (0.91, 3.78) | -5.36 | **<.001** | 1.94  (1.01, 3.77) | 2.15  (1.06, 4.19) | 1.65  (0.91, 3.21) | -2.18 | **0.029** |
| BMI | 29.50(24.90, 35.21) | 29.82(25.09, 35.60) | 28.70(24.67, 33.15) | -2.90 | **0.004** | 28.09 (23.69, 34.60) | 28.20(23.61, 34.24) | 28.01  (24.13, 35.27) | -0.08 | 0.935 |
| Mets | 180 (0, 1680) | 160(0, 1680) | 240(0, 1694) | -0.74 | 0.459 | 240(0,1800) | 240 (0, 1796) | 212(0,1791.17) | -0.45 | 0.653 |
| Number of pregnancies | 3 (2, 4) | 3 (2, 4.) | 3 (2, 4) | -0.82 | 0.415 | 3 (2, 4) | 2.21 (2, 3.98) | 3 (2, 4) | -3.26 | **0.001** |
| First sexual age | 18 (16, 20) | 18 (16, 20) | 17 (16, 19) | -3.72 | **<.001** | 17 (15, 18) | 17 (15, 19) | 16 (15, 18) | -2.50 | **0.012** |
| Number of sexual partners | 4 (2, 8) | 4 (2, 7) | 5 (3, 10) | -7.38 | **<.001** | 5 (2, 10) | 4 (2, 8) | 7 (3, 12) | -5.51 | **<.001** |
| Race |  |  |  | 19.35 | **<.001** |  |  |  | 52.06 | **<.001** |
| Mexican American | 249 (15.83) | 183 (15.93) | 66 (15.57) |  |  | 86 (18.49) | 61 (19.61) | 25 (16.23) |  |  |
| Other Hispanic | 179 (11.38) | 137 (11.92) | 42 (9.91) |  |  | 38 (8.17) | 24 (7.72) | 14 (9.09) |  |  |
| Non-Hispanic White | 693 (44.06) | 519 (45.17) | 174 (41.04) |  |  | 213 (45.81) | 162 (52.09) | 51 (33.12) |  |  |
| Non-Hispanic Black | 332 (21.11) | 213 (18.54) | 119 (28.07) |  |  | 99 (21.29) | 38 (12.22) | 61 (39.61) |  |  |
| Other Race | 120 (7.63) | 97 (8.44) | 23 (5.42) |  |  | 29 (6.24) | 26 (8.36) | 3 (1.95) |  |  |
| Education level |  |  |  | 5.47 | 0.065 |  |  |  | 4.85 | **0.028** |
| ≤high school | 712 (45.26) | 504 (43.86) | 208 (49.06) |  |  | 208 (44.73) | 128(41.16) | 80 (51.95) |  |  |
| ＞high school | 861 (54.74) | 645 (56.14) | 216 (50.94) |  |  | 257 (55.27) | 183 (58.84) | 74 (48.05) |  |  |
| Marital status |  |  |  | 65.86 | **<.001** |  |  |  | 30.36 | **<.001** |
| Married | 811 (51.56) | 663 (57.70) | 148 (34.91) |  |  | 231 (49.68) | 179 (57.56) | 52 (33.77) |  |  |
| Widowed | 82 (5.21) | 51 (4.44) | 31 (7.31) |  |  | 1 (0.22) | 1 (0.32) | 0 (0.00) |  |  |
| Divorced | 302 (19.20) | 195 (16.97) | 107 (25.24) |  |  | 55 (11.83) | 27 (8.68) | 28 (18.18) |  |  |
| Separated | 74 (4.70) | 49 (4.26) | 25 (5.90) |  |  | 23 (4.95) | 9 (2.89) | 14 (9.09) |  |  |
| Never married | 209 (13.29) | 128 (11.14) | 81 (19.10) |  |  | 109 (23.44) | 66 (21.22) | 43 (27.92) |  |  |
| Living with partner | 95 (6.04) | 63 (5.48) | 32 (7.55) |  |  | 46 (9.89) | 29 (9.32) | 17 (11.04) |  |  |
| Smoking status |  |  |  | 31.70 | **<.001** |  |  |  | 11.91 | **0.003** |
| never smoke | 887 (56.39) | 687 (59.79) | 200 (47.17) |  |  | 271 (58.28) | 198 (63.67) | 73 (47.40) |  |  |
| past smoke | 324 (20.60) | 238 (20.71) | 86 (20.28) |  |  | 71 (15.27) | 44 (14.15) | 27 (17.53) |  |  |
| current smoke | 362 (23.01) | 224 (19.50) | 138 (32.55) |  |  | 123 (26.45) | 69 (22.19) | 54 (35.06) |  |  |
| Hypertension |  |  |  | 2.02 | 0.155 |  |  |  | 1.35 | 0.246 |
| No | 994 (63.19) | 714 (62.14) | 280 (66.04) |  |  | 103 (22.15) | 64 (20.58) | 39 (25.32) |  |  |
| Yes | 579 (36.81) | 435 (37.86) | 144 (33.96) |  |  | 362 (77.85) | 247 (79.42) | 115 (74.68) |  |  |
| Female hormone use |  |  |  | 0.09 | 0.758 |  |  |  | 0.06 | 0.813 |
| No | 1302(82.77) | 949 (82.59) | 353 (83.25) |  |  | 407(87.53) | 273(87.78) | 134(87.01) |  |  |
| Yes | 271(17.23) | 200 (17.41) | 71 (16.75) |  |  | 58(12.47) | 38(12.22) | 20(12.99) |  |  |
| Diabetes |  |  |  | 0.51 | 0.477 |  |  |  | 0.06 | 0.806 |
| No | 1372 (87.22) | 998 (86.86) | 374 (88.21) |  |  | 29 (6.24) | 20 (6.43) | 9 (5.84) |  |  |
| Yes | 201 (12.78) | 151 (13.14) | 50 (11.79) |  |  | 436 (93.76) | 291 (93.57) | 145 (94.16) |  |  |

Continuous variables are presented as Median (Q1, Q3), categorical variables as n (%)

Z: Mann-Whitney test, χ²: Chi-square test
