# Supplementary material for: Association between sexually transmitted infections and reproductive lifespan: analysis of the NHANES 1999–2023
Source: BMC Public Health. 2026 May 22;26:2161. doi: 10.1186/s12889-026-27795-2 (PMC13374277; doi:10.1186/s12889-026-27795-2)
Supplement: Supplementary file 2 — Supplementary Material 2. [file 12889_2026_27795_MOESM2_ESM.docx]

***Table S2 Baseline characteristics of the BV participants***

| Characteristics | Total (n = 73) | Negative (n = 24) | Positive (n = 49) | Z/χ² | *P* |
| --- | --- | --- | --- | --- | --- |
|  |  |  |  |  |  |
| RLS | 29.00 (17.00, 33.00) | 29.50 (17.00, 34.00) | 28.00 (16.00, 33.00) | -0.31 | 0.760 |
| Age | 46.00 (31.00, 49.00) | 47.00 (30.75, 48.25) | 45.00 (36.00, 49.00) | -0.01 | 0.991 |
| PIR | 1.92 (1.08, 4.39) | 2.94 (1.44, 4.65) | 1.55 (1.08, 4.28) | -0.95 | 0.344 |
| BMI | 27.09 (24.30, 32.85) | 27.55 (24.82, 37.20) | 26.85 (24.30, 31.71) | -0.66 | 0.507 |
| Mets | 709.33 (112.00, 2038.00) | 942.33 (544.00, 2320.50) | 360.00 (84.00, 1577.33) | -2.11 | **0.035** |
| Number of pregnancies | 3.00 (2.00, 4.00) | 3.00 (2.00, 4.00) | 3.00 (1.00, 4.00) | -0.92 | 0.358 |
| First sexual age | 16.00 (15.00, 18.00) | 16.00 (15.00, 18.00) | 16.00 (15.00, 18.00) | -0.68 | 0.498 |
| Number of sexual partners | 5.00 (3.00, 10.00) | 5.00 (3.75, 10.00) | 6.00 (3.00, 10.00) | -0.22 | 0.827 |
| Race |  |  |  | - | 0.740 |
| Mexican American | 15 (20.55) | 4 (16.67) | 11 (22.45) |  |  |
| Other Hispanic | 4 (5.48) | 1 (4.17) | 3 (6.12) |  |  |
| Non-Hispanic White | 36 (49.32) | 12 (50.00) | 24 (48.98) |  |  |
| Non-Hispanic Black | 17 (23.29) | 6 (25.00) | 11 (22.45) |  |  |
| Other Race | 1 (1.37) | 1 (4.17) | 0 (0.00) |  |  |
| Educational level |  |  |  | 0.06 | 0.806 |
| ≤high school | 38 (52.05) | 12(50.00) | 26 (53.06) |  |  |
| ＞high school | 35 (47.95) | 12 (50.00) | 23 (46.94) |  |  |
| Marital status |  |  |  | - | 0.415 |
| Widowed | 32 (43.84) | 10 (41.67) | 22 (44.90) |  |  |
| Divorced | 1 (1.37) | 1 (4.17) | 0 (0.00) |  |  |
| Separated | 9 (12.33) | 4 (16.67) | 5 (10.20) |  |  |
| Never married | 3 (4.11) | 2 (8.33) | 1 (2.04) |  |  |
| Living with partner | 20 (27.40) | 5 (20.83) | 15 (30.61) |  |  |
| Never married | 8 (10.96) | 2 (8.33) | 6 (12.24) |  |  |
| Smoking status |  |  |  | 0.33 | 0.846 |
| never smoke | 30 (41.10) | 9 (37.50) | 21 (42.86) |  |  |
| past smoke | 13 (17.81) | 4 (16.67) | 9 (18.37) |  |  |
| current smoke | 30 (41.10) | 11 (45.83) | 19 (38.78) |  |  |
| Hypertension |  |  |  | 0.00 | 0.962 |
| No | 55 (75.34) | 18 (75.00) | 37 (75.51) |  |  |
| Yes | 18 (24.66) | 6 (25.00) | 12 (24.49) |  |  |
| Female hormone use |  |  |  | 0.00 | 0.958 |
| No | 52(71.23) | 17(70.83) | 35(71.43) |  |  |
| Yes | 21(28.77) | 7(29.17) | 14(28.57) |  |  |
| Diabetes |  |  |  | - | 1.000 |
| No | 70 (95.89) | 23 (95.83) | 47 (95.92) |  |  |
| Yes | 3 (4.11) | 1 (4.17) | 2 (4.08) |  |  |
| Continuous variables are presented as Median (Q1, Q3), categorical variables as n (%)  Z: Mann-Whitney test, χ²: Chi-square test, -: Fisher exact | | | | | |
|  | | | | | |
