# Supplementary material for: Association between sexually transmitted infections and reproductive lifespan: analysis of the NHANES 1999–2023
Source: BMC Public Health. 2026 May 22;26:2161. doi: 10.1186/s12889-026-27795-2 (PMC13374277; doi:10.1186/s12889-026-27795-2)
Supplement: Supplementary file 3 — Supplementary Material 3. [file 12889_2026_27795_MOESM3_ESM.docx]

***Table S3 Baseline characteristics of the TV participants***

| Characteristics | Total (n = 68) | Negative  (n = 62) | Positive  (n = 6) | Z/χ² | *P* |
| --- | --- | --- | --- | --- | --- |
|  |  |  |  |  |  |
| RLS | 29.00  (17.00, 33.00) | 28.50  (17.00, 33.00) | 33.50  (30.00, 34.00) | -1.56 | 0.119 |
| Age | 46.00  (33.25, 49.00) | 46.00  (31.75, 49.00) | 47.50  (44.75, 48.00) | -0.69 | 0.491 |
| PIR | 1.91  (1.06, 4.17) | 2.13  (1.09, 4.28) | 1.20  (0.64, 1.47) | -1.89 | 0.058 |
| BMI | 27.54  (24.35, 32.93) | 27.38  (24.26, 33.08) | 27.54  (25.80, 27.91) | -0.01 | 0.991 |
| Mets | 730.33(112.00, 2060.50) | 739.67(140.00, 2105.50) | 133.00 (10.50, 674.00) | -1.16 | 0.246 |
| Number of pregnancies | 3.00 (2.00, 4.00) | 3.00 (2.00, 4.00) | 3.50(3.00, 8.50) | -1.08 | 0.279 |
| First sexual age | 16.00  (15.00, 18.00) | 16.00  (15.00, 18.00) | 15.00  (14.25, 15.00) | -1.86 | 0.063 |
| Number of sexual partners, | 5.00  (3.00, 10.00) | 5.00  (3.00, 10.00) | 4.50  (3.00, 12.00) | -0.03 | 0.974 |
| Race |  |  |  | - | 0.908 |
| Mexican American | 12 (17.65) | 11 (17.74) | 1 (16.67) |  |  |
| Other Hispanic | 4 (5.88) | 4 (6.45) | 0 (0.00) |  |  |
| Non-Hispanic White | 35 (51.47) | 32 (51.61) | 3 (50.00) |  |  |
| Non-Hispanic Black | 16 (23.53) | 14 (22.58) | 2 (33.33) |  |  |
| Other Race | 1 (1.47) | 1 (1.61) | 0 (0.00) |  |  |
| Educational level |  |  |  | 4.90 | **0.027** |
| ≤ high school | 33 (48.53) | 27 (43.55) | 6 (100.00) |  |  |
| ＞high school | 35 (51.47) | 35 (56.45) | 0 (0.00) |  |  |
| Marital status |  |  |  | - | 0.085 |
| Widowed | 31 (45.59) | 30 (48.39) | 1 (16.67) |  |  |
| Divorced | 1 (1.47) | 1 (1.61) | 0 (0.00) |  |  |
| Separated | 9 (13.24) | 8 (12.90) | 1 (16.67) |  |  |
| Never married | 3 (4.41) | 2 (3.23) | 1 (16.67) |  |  |
| Living with partner | 18 (26.47) | 17 (27.42) | 1 (16.67) |  |  |
| Never married | 6 (8.82) | 4 (6.45) | 2 (33.33) |  |  |
| Smoking status |  |  |  | - | 0.244 |
| never smoke | 28 (41.18) | 26 (41.94) | 2 (33.33) |  |  |
| past smoke | 14 (20.59) | 14 (22.58) | 0 (0.00) |  |  |
| current smoke | 26 (38.24) | 22 (35.48) | 4 (66.67) |  |  |
| Hypertension |  |  |  | 0.00 | 1.000 |
| No | 51 (75.00) | 47 (75.81) | 4 (66.67) |  |  |
| Yes | 17 (25.00) | 15 (24.19) | 2 (33.33) |  |  |
| Female hormone use |  |  |  | 0.01 | 0.932 |
| No | 50(73.53) | 45(72.58) | 5(83.33) |  |  |
| Yes | 18(26.47) | 17(27.42) | 1(16.67) |  |  |
| Diabetes |  |  |  | - | 1.000 |
| No | 64 (94.12) | 58 (93.55) | 6 (100.00) |  |  |
| Yes | 4 (5.88) | 4 (6.45) | 0 (0.00) |  |  |
| Continuous variables are presented as Median (Q1, Q3), categorical variables as n (%)  Z: Mann-Whitney test, χ²: Chi-square test, -: Fisher exact | | | | | |
|  | | | | | |
