# Supplementary material for: Association between sexually transmitted infections and reproductive lifespan: analysis of the NHANES 1999–2023
Source: BMC Public Health. 2026 May 22;26:2161. doi: 10.1186/s12889-026-27795-2 (PMC13374277; doi:10.1186/s12889-026-27795-2)
Supplement: Supplementary file 4 — Supplementary Material 4. [file 12889_2026_27795_MOESM4_ESM.docx]

***Table S4 Baseline characteristics of the CT participants***

| Characteristics | Total (n = 200) | Negative  (n = 199) | Positive  (n = 1) | Z/χ² | *P* |
| --- | --- | --- | --- | --- | --- |
|  |  |  |  |  |  |
| RLS | 13.00  (8.00, 17.00) | 13.00  (8.00, 17.00) | 5.00  (5.00, 5.00) | -1.44 | 0.150 |
| Age | 28.00  (23.00, 33.00) | 28.00  (23.00, 33.00) | 24.00  (24.00, 24.00) | -0.71 | 0.477 |
| PIR | 1.54  (0.84, 2.89) | 1.53  (0.83, 2.85) | 2.96  (2.96, 2.96) | -0.87 | 0.386 |
| BMI | 26.23  (21.46, 32.47) | 26.26  (21.59, 32.48) | 16.02  (16.02, 16.02) | -1.65 | 0.100 |
| Mets | 300.00(0.00, 2400.00) | 300.00 (0.00, 2400.00) | 0.00  (0.00, 0.00) | -1.07 | 0.283 |
| Number of pregnancies | 2.00  (1.00, 3.00) | 2.00  (1.00, 3.00) | -0.13  (-0.13, -0.13) | -1.72 | 0.085 |
| First sexual age | 16.00  (15.00, 18.00) | 16.00  (15.00, 18.00) | 16.00  (16.00, 16.00) | -0.05 | 0.958 |
| Number of sexual partners | 5.00  (2.00, 10.00) | 5.00  (2.00, 10.00) | 8.00  (8.00, 8.00) | -0.57 | 0.566 |
| Race |  |  |  | - | 1.000 |
| Mexican American | 31 (15.50) | 31 (15.58) | 0 (0.00) |  |  |
| Other Hispanic | 15 (7.50) | 15 (7.54) | 0 (0.00) |  |  |
| Non-Hispanic White | 97 (48.50) | 96 (48.24) | 1 (100.00) |  |  |
| Non-Hispanic Black | 40 (20.00) | 40 (20.10) | 0 (0.00) |  |  |
| Other Race | 17 (8.50) | 17 (8.54) | 0 (0.00) |  |  |
| Educational level |  |  |  | - | 1.000 |
| ≤ high school | 88 (44.00) | 88 (44.22) | 0 (0.00) |  |  |
| ＞high school | 112 (56.00) | 111 (55.78) | 1 (100.00) |  |  |
| Marital status |  |  |  | - | 0.595 |
| Widowed | 81 (40.50) | 81 (40.70) | 0 (0.00) |  |  |
| Divorced | 12 (6.00) | 12 (6.03) | 0 (0.00) |  |  |
| Separated | 5 (2.50) | 5 (2.51) | 0 (0.00) |  |  |
| Never married | 71 (35.50) | 70 (35.18) | 1 (100.00) |  |  |
| Living with partner | 31 (15.50) | 31 (15.58) | 0 (0.00) |  |  |
| Smoking status |  |  |  | - | 0.370 |
| never smoke | 126 (63.00) | 126 (63.32) | 0 (0.00) |  |  |
| past smoke | 20 (10.00) | 20 (10.05) | 0 (0.00) |  |  |
| current smoke | 54 (27.00) | 53 (26.63) | 1 (100.00) |  |  |
| Hypertension |  |  |  | - | 1.000 |
| No | 177 (88.50) | 176 (88.44) | 1 (100.00) |  |  |
| Yes | 23 (11.50) | 23 (11.56) | 0 (0.00) |  |  |
| Female hormone use |  |  |  | - | 1.000 |
| No | 195 (97.50) | 194 (97.49) | 1 (100.00) |  |  |
| Yes | 5 (2.50) | 5 (2.51) | 0 (0.00) |  |  |
| Diabetes |  |  |  | - | 1.000 |
| No | 197 (98.50) | 196 (98.49) | 1 (100.00) |  |  |
| Yes | 3 (1.50) | 3 (1.51) | 0 (0.00) |  |  |
| Continuous variables are presented as Median (Q1, Q3), categorical variables as n (%)  Z: Mann-Whitney test, χ²: Chi-square test, -: Fisher exact | | | | | |
